# Technological, functional and safety properties of lactobacilli isolates from soft wheat sourdough and their potential use as antimould cultures

**DOI:** 10.1007/s11274-021-03114-2

**Published:** 2021-08-07

**Authors:** Jatziri Mota-Gutierrez, Irene Franciosa, Marianna Ruggirello, Paola Dolci

**Affiliations:** Department of Agricultural, Forest and Food Sciences, University of Turin, Largo Paolo Braccini 2, 10095 Grugliasco, Turin, Italy

**Keywords:** Fungi, Lactic acid bacteria, *Lactiplantibacillus plantarum*, *Lacticaseibacillus casei*, Wheat, Antifungal activity

## Abstract

Bakery products are a common medium for fungal growth due to their high-water activity and nutrients availability. The application of lactic acid bacteria (LAB) isolated from wheat bran or other cereals has shown great potential in controlling the growth of spoilage fungi, guarantee quality and prolong the shelf life of bakery products. This study outlines the antifungal, technological, functional and safety properties of autochthonous LAB microbiota isolated from type 0 soft wheat sourdough fermentation. Antifungal activity of 77 LAB belonging to *Lactiplantibacillus plantarum* and *Lacticaseibacillus casei* species isolated from spontaneous sourdough fermentation was tested *in vitro* against 16 spoilage fungi. Our findings demonstrated that the antifungal activity, enzymatic and safety properties of LAB isolates vary strain-dependently. Four LAB isolates (*Lp. plantarum* A16, A25, B11, and B15) showed the best traits, in particular strong antifungal activity and good capabilities to produce exopolysaccharides from different carbon sources *in vitro*. Care should be taken when using *Lp. plantarum* A310 and B18 and *Lc. casei* A23, as starter cultures, since these isolates exhibited a multiple antibiotic-resistance. Here we showed the promising potential of different LAB isolates as bio-preservative agents and to provide new insights regarding their prospective use as starter cultures to guarantee safety and palatability.

**Graphic abstract:**

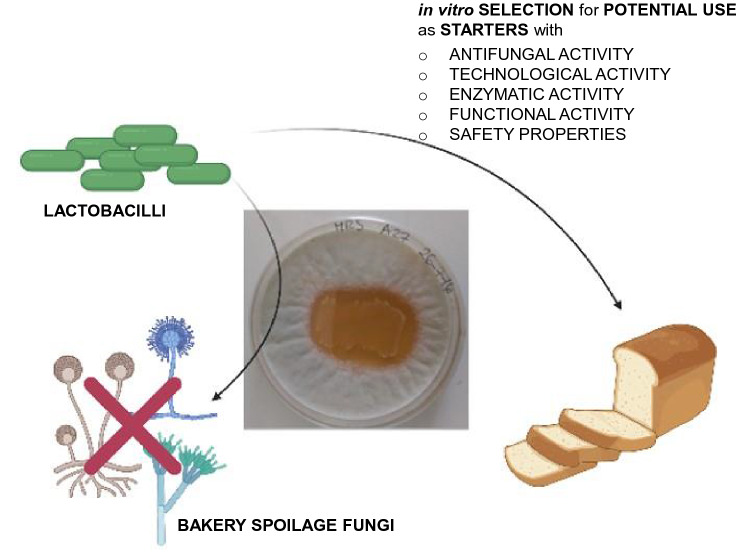

## Introduction

Cereals and cereal products, such as bread, are considered a staple food. The high content in carbohydrates, dietary fiber and protein sources of bread, makes this cereal product one of the main dietary sources for human nutrition but also a desirable substrate for mold contaminants. The contamination sources of bakery products are mostly environmentally based, coming from the air, handling, processing equipment and storage, as well as from the flour itself (Rosenkvist and Hansen 1995; Reale et al. 2013; Garcia et al. 2019). *Aspergillus, Penicillium, Fusarium, Rhizopus* and *Mucor* are the most common mold genera associated with bakery product spoilage (Legan 1993; Garcia et al. 2019). Fungi are responsible for off-flavors and production of mycotoxins which belong to the most toxic contaminants occurring in a wide range of food commodities (Bennet and Wallsgrove 1994).

Research in the field of characterization and selection of natural bio-protective agents that also guarantees quality and ensure safety is becoming increasingly important to promote the use of eco-friendly alternatives instead of using chemical additives in the food industry. The use of *Lactiplantibacillus plantarum* strains as natural bio-protective agents has emerged as a sustainable alternative for food conservation. The antifungal, technological, safety and nutritional properties of *Lp. plantarum* strains such as broad-range activity towards spoilage fungi, high stability at various pH and temperatures, and production of bacteriocins have been extensively studied (De Angelis et al. 2003; Gupta and Srivastava 2014; Manini et al. 2016; Ruiz Rodríguez et al. 2016; Arena et al. 2019). Besides *Lp. plantarum, Levilactobacillus brevis, Limosilactobacillus reuteri, Furfurilactobacillus rossiae* strains isolated from sourdough have been demonstrated to control mold growth and the production of aflatoxin, as well as showing technological and enzymatic activities (Garofalo et al. 2012; Manini et al. 2016; Sadeghi et al. 2019a, b).

The technological and functional characteristics of LAB strains used in the food chain contribute to the sensory quality of food products (De Vuyst et al. 2010; Viana de Souza and Silva Dias 2017). In particular, LAB are able to enrich dough, producing exopolysaccharides (EPS), enzymes, organic acids and antimicrobial compounds (Torrieri et al. 2014). In this regards, the characterization of LAB strains isolated mainly from wheat germ, wheat bran, and hard wheat has been extensively investigated (Minervini et al. 2010; Garofalo et al. 2012; Manini et al. 2016; Sadeghi et al. 2019a, b; Fekri et al. 2020). Whereas somehow the characterization of LAB strains and the identification of the microbial composition of soft wheat is rarely investigated (Nionelli et al. 2014; Taccari et al. 2016). Soft wheat is *per capita* the main grain consumed, followed by maize and rye (Statistik Austria, 2021). The high consumption of wheat is linked to the vast number of products containing wheat as the main ingredient such as bread, pasta, other bakery products and is used for thickening gravy and sauces. In this study, LAB isolates were chosen from soft wheat sourdough for its nutritional characteristics, such as less starch and gluten content compared with bran wheat (USDA, 2020). Despite many studies, it is still unclear to what extent the sourdough microbiota is affected and selected by the type of flour used. For sure, cereal fermentation processes depend on specific determinants, and the type of flour is one of the most important (Nionelli et al. 2014; Taccari et al. 2016).

The unique characteristics of the different types of sourdough influence also the microbial ecosystem (Gänzle and Zheng 2019). In spontaneous sourdough fermentation, LAB such as *Lp. plantarum, Lacticaseibacillus casei, Fl. rossiae*, and pediococci, dominate the microbial ecosystem (Gänzle and Zheng 2019; Minervini et al. 2010). In bakery industry, different LAB species have been used as a starter culture or co-culture to improve the sensorial attributes and direct microbial development (Salmenkallio-Marttila et al., 2001). Although LAB have been applied for decades in food preservation, leading to their classification as Generally Recognized As Safe (GRAS), there is growing evidence that *Lp. plantarum, Ll. brevis* and *Latilactobacillus curvatus* isolated from sourdough can be resistant to antibiotics such as clindamycin (Manini et al. 2016). In this research, we studied autochthonous LAB strains previously isolated from spontaneous fermented sourdough-like soft wheat flour for their antifungal activity to explore their bioprotective potential. Thus, the best performing LAB isolates were characterized for their technological, functional and safety properties to provide new insights towards the potential use of new LAB isolates as starter cultures in food applications, such as wheat fermentation and sourdough bread, without compromising food safety.

## Materials and methods

### LAB isolate selection based on antifungal activity

In total, seventy-seven LAB cultures, identified as *Lp. plantarum* (*n* = 68) and *Lc. casei* (*n* = 9) by species-specific PCR (Berthier and Ehrlich, 1998) and 16 S rRNA gene sequencing (Teymoortash et al. 2002), respectively, were revived from glycerol stock stored at −80 °C at DISAFA Collection (Department of Agricultural, Forest, and Food Sciences, University of Turin), in De Man, Rogosa and Sharpe (MRS, Oxoid, Milan, Italy) broth twice and incubated at 30 °C for 24 h. LAB strains had been previously isolated from soft wheat sourdoughs produced in both artisanal and industrial bakeries located in northwest Italy. Precisely, LAB strains coded with “A” came from a company (located in Piedmont region) producing the traditional Italian products Colomba and Panettone; LAB strains coded with “B” from an artisanal bakery (located in Piedmont region) mainly producing bread; and with “C” from an artisanal bakery (located in Aosta Valley region) producing Panettone. Sixteen mold strains were revived in Malt Extract Agar (Oxoid) at 27 °C for 5 days. They had been previously isolated from bakery products (moldy bread and Panettone) and stored at DISAFA Collection or purchased from the Mycotheca Universitatis Taurinenesis (MUT) (Department of Life Sciences and Systems Biology, University of Turin) and the Federal Research Centre for Nutrition and Foods (BFE) (Karlsruhe, Germany) (Table 1).

**Table 1:** Antifungal activity of the 20 selected LAB strains isolated from soft wheat fermentations against fungal strains recurrent in bakery product spoilage

LAB species		*A. fumigatus*	*A. niger*	*P. expansum*	*P. chrysogenum*	*P. nordicum BFE 536*	*P. funiculosum*	*P. globosum*	*P. aurantiogriseum*	*P. nalgiovense*	*P. citrinum*	*P. MUT 2546*	* A. flavus MUT 3247*	* A. flavus*	*M. variosporus*	*M. racemosus*	*M. plumbeus*
A16	*Lp. plantarum*	++	++	++	−	++	++	+	++	++	++	−	++	++	−	+	++
A23	*Lc. casei*	++	++	++	+	++	++	++	+	+	−	−	+	++	−	−	+
A25	*Lp. plantarum*	++	++	++	+	++	++	++	++	+	+	−	+	+	−	++	++
A310	*Lp. plantarum*	++	++	++	−	++	++	+	+	+	++	−	++	++	−	++	++
B11	*Lp. plantarum*	++	++	++	−	++	++	+	++	−	++	−	+	++	−	+	++
B15	*Lp. plantarum*	++	++	++	−	++	++	+	+	−	++	−	+	+	−	+	++
B18	*Lp. plantarum*	++	++	++	+	++	++	+	+	+	++	−	−	+	−	−	++
B19	*Lp. plantarum*	++	++	++	−	++	++	++	+	−	−	−	−	−	−	−	−
B21	*Lp. plantarum*	++	++	++	−	++	++	++	++	+	−	−	−	+	−	−	+
B23	*Lp. plantarum*	++	++	++	−	++	++	++	+	+	−	−	−	−	−	+	++
B27	*Lp. plantarum*	++	++	++	−	++	++	+	++	−	++	−	+	+	−	+	++
B28	*Lc. casei*	++	+	++	−	++	++	−	+	−	++	−	+	−	−	+	++
B29	*Lp. plantarum*	++	++	++	−	++	++	−	−	−	−	−	−	−	−	−	−
B32	*Lp. plantarum*	++	++	++	−	++	++	++	+	+	++	−	++	+	−	−	++
B33	*Lc. casei*	++	++	++	−	++	++	−	+	++	−	−	−	++	−	−	++
C11	*Lp. plantarum*	++	++	++	−	++	++	−	−	−	−	−	+	++	−	−	++
C12	*Lp. plantarum*	++	++	++	+	++	++	−	+	+	+	−	−	−	−	+	++
C17	*Lp. plantarum*	++	++	++	−	++	++	++	+	+	++	−	−	−	−	+	++
C28	*Lp. plantarum*	++	++	++	+	++	++	++	−	+	−	−	−	+	−	+	++
C38	*Lp. plantarum*	++	++	++	−	++	++	−	+	−	++	−	+	−	−	+	++

Interpretation of inhibition zone − = no inhibition; + = moderate inhibition; ++ = complete inhibition

*MUT* strains from Mycotheca Universitatis Taurinenesis, *BFE* strains from Federal Research Centre for Nutrition and Foods, all the other strains are from DISAFA Collection

The seventy-seven LAB cultures were preliminarily screened for their antifungal activity against three fungal strains selected in this study as main targets (*Aspergillus fumigatus, Aspergillus niger* and *Penicillium expansum*). Twenty LAB isolates (*Lp. plantarum* = 17 and *Lc. casei* = 3) were preselected for their stronger antifungal activity against the fungal target cultures and further tested against the rest of the molds (*n* = 13).

The antifungal activity of LAB isolates was determined using the overlay method described by Magnusson and Schnürer with some modifications (Magnusson and Schnürer 2001). From Malt Extract Agar plates, the conidia were scraped and harvested from mycelium surface with Ringer solution (Oxoid) and counted by means of Burker chamber. LAB isolates were inoculated in two 2-cm-long lines on MRS agar plates and incubated at 37 °C for 24 h. The plates were then overlaid with 10 mL of Malt Extract Soft Agar (Biolife, Milan, Italy) inoculated with 10^4^ conidia/mL of the fungal suspension. The overlaid plates were incubated at 25 °C for 5 days. Plates were then examined for clear zones of inhibition around the LAB streaks and scored as − (no growth suppression), + (medium growth suppression) and ++ (total growth suppression). Inhibition tests were done in triplicates.

### Growth and acidification rate of selected LAB isolates

The microbial counts, pH and Total Titratable Acidity (TTA) were analyzed to determine the fermentation capacity of the twenty pre-selected LAB isolates following Manini et al. (2016) protocols with some modifications. An overnight culture (1% v/v) of each LAB isolate was individually inoculated into a sample (100 g) of type “0” soft wheat flour (15% w/v of flour and 85% of water) and incubated for 7 h at 30 °C on a horizontal shaker Promax 2020 (Heidolph, Schwabach, Germany). Classical microbiological analysis was performed immediately after LAB inoculum and after 7 h of fermentation. MRS agar was used for counting lactobacilli incubated at 30 °C for 48–72 h. The dynamics of pH and TTA were measured after inoculum and during the fermentation at 4 and 7 h. The kinetics of acidification by LAB isolates were measured from the suspension of 10 g sample of sourdough in 100 mL of deionized water. For the assessment of TTA, this suspension was titrated with 0.1 M NaOH to a final pH of 8.5. Results of TTA were expressed as milliliters of 0.1 M NaOH needed to achieve the final pH of 8.5. All samples were analyzed in triplicate.

### Technological, functional and safety properties of selected LAB isolates

#### Exopolysaccharide (EPS) production

Overnight LAB cultures were plated on different MRS agar media with glucose, sucrose, maltose, lactose, and starch as the only carbon sources. Plates were incubated at 30 °C for 48 h. Triplicate plates of each LAB were scored for mucoid properties (scale of ++ = excess EPS, + = moderate EPS and − = no visible mucoid). Colonies were scored as positive if strings were detected when the colony was touched once with an inoculating loop (Manini et al. 2016).

#### Xylanase activity

To test xylanase producing microorganisms at different pH, two different agar media were prepared by adding 0.1% (w/v) of the dyed substrate (Remazolbrilliant Blue R plus Azo-Xylan – birchwood) to a sodium phosphate buffer, 100 mM, pH 6 and to a sodium acetate buffer, 100 mM, pH 4.5 (Manini et al. 2016). Triplicate plates were inoculated with 2 µL of the overnight LAB cultures and incubated at 30 °C for 48 h. The xylanase activity was determined based on the diameter of the clearing zone of inhibition around the LAB streaks.

#### Amylolytic activity

The starch degradation by spot inoculation of LAB isolates was evaluated in triplicates, as described by Rodriguez et al. (2016) with some modifications. LAB isolates (2 µL) were streaked on plates containing MRS-starch medium in which glucose was replaced by starch (1%). Inoculated plates were incubated at 30 °C for 48 h and then stored at 4 °C for 24 h. After 72 h of incubation, plates were flooded with iodine solution (4%). Amylase production was indicated by a clear zone around the colonies, while the rest of the plate stained blue-black. *Amylolactobacillus amylophilus* 20,533 was used as a positive control strain. The amylolytic activity was determined based on the diameter of the clearing zone of inhibition around the LAB streaks.

#### Phytase activity

LAB isolates preliminary inoculated in MRS broth and incubated at 37 °C for 24 h were then growth at 30 °C for 48 h in modified Chalmers broth with 1% neutral red and 1% calcium phytate (Manini et al. 2016). Phytase activity was then determined based on the diameter of the clearing zone around the holes in the modified Chalmers agar plates. To eliminate false positive results, caused by microbial acid production, Chalmer agar plates were flooded twice with 2% (w/v) aqueous cobalt chloride solution. After 20 min, the solution was removed and phytase activity evaluated (Anastasio et al. 2010).

#### Proteolytic activity

Proteolysis of LAB cultures was assayed against gelatin or casein on agar plates prepared as follows: 2% sucrose, 0.5% yeast extract, 2% peptone, 1.5% agar autoclaved and supplemented with 1 % gelatin or casein, and incubated at 37 °C for 48 h. Extracellular protease activity was detected after staining agar plates with Coomassie blue (0.25%, w/v) for 1 h in methanolacetic acid-water 5:1:4 (v/v/v) and destaining with methanolacetic acid-water. Areas of enzymatic activity were scored as grade –, when no visible halo was present; +, when visible proteolysis was limited to 1–2 mm around the colony; and ++, when the zone of proteolysis was more than 2 mm from the colony.

#### Antibiotic resistance

The minimum inhibitory concentration (MIC) of eight antibiotics (ampicillin-Amp, gentamicin-Gen, kanamycin-Kan, streptomycin-Str, erythromycin-Ery, clindamycin-Cli, tetracycline-Tet, chloramphenicol-Cho) was determined by the microdilution method as reported by the ISO 10,932:2010 standard method (ISO 10,932/IDF 223, 2010). Seven LAB isolates, selected according to their strong antifungal activity, EPS production and enzymatic activity, were classified as resistant/sensitive according to the cut-off values defined according to the FEEDAP Panel (EFSA-FEEDAP^2012^).

### Statistical analysis

The results are presented as mean values ± standard deviation of triplicate measurements. Statistical analyses were performed using generalized linear mixed-effects model for non-distributed data set. Mixed models were chosen for their ability to capture both fixed (LAB isolates) and random effects (number of replicates). When no dispersion of the data distribution was observed (EPS production, proteolytic activity, antifungal activity) any statistical test was used. The *P*-values were adjusted using Bonferroni’s method; when the linear mixed model revealed significant differences (*P* < 0.05) the Duncan honestly significant difference (HSD) test was applied. Mixed models were built and evaluated following Crawley (Crawley 2007), using R version 3.3.2.

## Results

### LAB isolates showed different inhibitory activity against fungal species

The antifungal assays showed that all the 20 selected LAB isolates were able to inhibit *A. fumigatus, A. niger, P. expansum, Penicillium nordicum*, and *Penicillium funiculosum*. Overall, LAB isolates were capable of inhibiting the growth of 5 up to 13 fungal species (Table 1). *Lp. plantarum* A16, followed by A310 demonstrated strong antifungal activity against 11 and 10 fungal species, respectively, whereas *Lp. plantarum* B29 showed strong antifungal activity against only 5 fungal species. In contrast, none of LAB isolates were able to inhibit the growth of *Mucus variosporus* and *Penicillium* sp. *MUT* 2546 (Table 1).

### Significant differences between growth and pH rate of LAB isolates

The growth, in sourdough medium, of all the tested LAB isolates increased substantially after 7 h of incubation (average increase approximately 1.47 log CFU/g, Table 2). A significant difference in the growth rate, pH values, and TTA was observed among LAB isolates (Table 2). In detail, significantly higher loads of *Lp. plantarum* B27 were observed at the beginning and the end of the fermentation (9.20 and 11.06 log CFU/g, respectively), while *Lp. plantarum* A310 and B11 showed the lowest load at the end of the fermentation (8.26 and 8.38 log CFU/g, respectively, *P* < 0.05 Table 2). Concerning pH value changes, we observed significantly higher pH values for *Lc. casei* A23 (5.19) and lowest values for *Lp. plantarum* A16, A25, B29, B32, C11 (4.15, 4.15, 4.15, 4.19 and 4.14, respectively, *P* < 0.05, Table 2) at the end of the fermentation. In parallel, significantly higher total acidity was observed for *Lp. plantarum* B29, C11 and C12 (2.20, 2.38 and 2.18, respectively), while significantly lower acidity was reported for *Lp. plantarum* B27 and *Lc. casei* A23 (1.08 and 0.65, respectively, *P* < 0.05, Table 2) at the end of the fermentation.

**Table 2:** LAB viable cell-counts (log CFU/g), pH and total titratable acidity variations in 7 h sourdough fermentation

LAB isolate			0 h				4 h				7 h				Dynamics
*Lp. plantarum*	**A16**														
		pH	6.60	±	0.07	abc	5.63	±	0.06	c	4.15	±	0.01	c	2.45
		TTA	0.35	±	0.07	abc	0.63	±	0.04		1.93	±	0.04	abc	−1.58
		log CFU/g	7.99	±	0.01	bc	nd				9.72	±	0.02	ab	1.73
*Lc. casei*	**A23**														
		pH	6.78	±	0.03	ab	6.21	±	0.09	ab	5.19	±	0.04	a	1.59
		TTA	0.20	±	0.00	bcde	0.38	±	0.04		0.65	±	0.42	c	−0.22
		log CFU/g	7.62	±	0.17	c	nd				9.34	±	0.15	b	1.72
*Lp. plantarum*	**A25**														
		pH	6.63	±	0.00	abc	5.45	±	0.15	c	4.15	±	0.01	c	2.48
		TTA	0.30	±	0.00	abcd	0.65	±	0.07		1.70	±	0.14	abc	−1.40
		log CFU/g	7.49	±	0.13	bc	nd				8.87	±	0.04	ab	1.38
*Lp. plantarum*	**A310**														
		pH	6.86	±	0.00	a	6.37	±	0.01	ab	4.42	±	0.11	abc	2.44
		TTA	0.15	±	0.00	e	0.28	±	0.04		1.63	±	0.25	abc	−1.48
		log CFU/g	7.31	±	0.02	bc	nd				8.26	±	0.21	bc	0.95
*Lp. plantarum*	**B11**														
		pH	6.80	±	0.01	abc	6.55	±	0.02	ab	4.60	±	0.11	abc	2.20
		TTA	0.18	±	0.04	de	0.23	±	0.04		1.20	±	0.07	abc	−1.20
		log CFU/g	7.33	±	0.04	bc	nd				8.30	±	0.06	c	0.97
*Lp. plantarum*	**B15**														
		pH	6.72	±	0.09	abc	5.74	±	0.09	abc	4.21	±	0.01	abc	2.51
		TTA	0.18	±	0.04	de	0.50	±	0.07		1.50	±	0.00	abc	−1.32
		log CFU/g	7.13	±	0.07	c	nd				8.47	±	0.01	ab	1.34
*Lp. plantarum*	**B18**														
		pH	6.72	±	0.04	abc	5.82	±	0.11	abc	4.24	±	0.08	abc	2.48
		TTA	0.15	±	0.00	e	0.45	±	0.07		1.53	±	0.11	abc	−1.38
		log CFU/g	7.36	±	0.03	bc	nd				8.37	±	0.13	ab	1.01
*Lp. plantarum*	**B19**														
		pH	6.64	±	0.02	abc	6.17	±	0.47	ab	4.51	±	0.41	abc	2.13
		TTA	0.25	±	0.00	abcde	0.38	±	0.18		1.40	±	0.35	abc	−1.15
		log CFU/g	7.37	±	0.13	bc	nd				8.31	±	0.28	bc	0.94
*Lp. plantarum*	**B21**														
		pH	6.72	±	0.04	abc	6.00	±	0.11	ab	4.24	±	0.03	abc	2.48
		TTA	0.23	±	0.04	bcde	0.43	±	0.04		1.65	±	0.00	abc	−1.42
		log CFU/g	7.41	±	0.02	bc	nd				8.38	±	0.00	c	0.97
*Lp. plantarum*	**B23**														
		pH	6.63	±	0.06	abc	5.67	±	0.01	abc	4.35	±	0.02	abc	2.28
		TTA	0.20	±	0.00	cde	0.55	±	0.00		1.48	±	0.04	abc	−1.28
		log CFU/g	7.34	±	0.48	bc	nd				8.52	±	0.01	ab	1.18
*Lp. plantarum*	**B27**														
		pH	6.76	±	0.03	abc	6.59	±	0.01	a	4.68	±	0.05	abc	2.08
		TTA	0.30	±	0.00	abcd	0.33	±	0.04		1.08	±	0.04	c	−0.78
		log CFU/g	9.20	±	0.00	a	nd				11.06	±	0.01	a	1.86
*Lc. casei*	**B28**														
		pH	6.63	±	0.05	abc	5.97	±	0.04	abc	4.64	±	0.13	abc	1.99
		TTA	0.33	±	0.04	abc	0.53	±	0.04		1.25	±	0.14	bc	−0.92
		log CFU/g	7.84	±	0.07	ab	nd				9.84	±	0.01	ab	2.00
*Lp. plantarum*	**B29**														
		pH	6.56	±	0.04	c	5.69	±	0.07	abc	4.15	±	0.07	c	2.41
		TTA	0.35	±	0.00	ab	0.50	±	0.00		2.18	±	0.39	a	−0.90
		log CFU/g	7.72	±	0.12	abc	nd				9.59	±	0.23	ab	1.87
*Lp. plantarum*	**B32**														
		pH	6.60	±	0.05	bc	5.70	±	0.07	abc	4.19	±	0.01	c	2.41
		TTA	0.35	±	0.07	ab	0.63	±	0.18		1.83	±	0.11	ab	−1.48
		log CFU/g	7.63	±	0.04	abc	nd				8.37	±	0.16	ab	0.74
*Lc. casei*	**B33**														
		pH	6.61	±	0.01	abc	5.98	±	0.09	ab	4.82	±	0.08	abc	1.79
		TTA	0.25	±	0.00	abcde	0.53	±	0.18		1.23	±	0.46	abc	−0.98
		log CFU/g	7.69	±	0.01	abc	nd				9.44	±	0.13	ab	1.75
*Lp. plantarum*	**C11**														
		pH	6.55	±	0.00	c	5.64	±	0.08	abc	4.14	±	0.01	c	2.41
		TTA	0.35	±	0.00	ab	0.60	±	0.00		2.20	±	0.35	a	−1.85
		log CFU/g	7.74	±	0.10	abc	nd				9.47	±	0.13	ab	1.73
*Lp. plantarum*	**C12**														
		pH	6.65	±	0.01	abc	6.05	±	0.02	ab	4.26	±	0.07	abc	2.39
		TTA	0.40	±	0.00	a	0.48	±	0.04		2.38	±	0.04	a	−1.98
		log CFU/g	7.93	±	0.02	abc	nd				9.40	±	1133.00	ab	1.47
		*Growth log CFU/g*									*1.47*				
*Lp. plantarum*	**C17**														
		pH	6.63	±	0.03	abc	6.37	±	0.01	ab	4.72	±	0.23	abc	1.91
		TTA	0.40	±	0.00	a	0.45	±	0.00		1.38	±	0.11	abc	−0.98
		log CFU/g	7.75	±	0.07	abc	nd				9.89	±	0.21	ab	2.14
		*Growth log CFU/g*									*2.14*				
*Lp. plantarum*	**C28**														
		pH	6.81	±	0.02	abc	6.07	±	0.01	ab	4.53	±	0.18	abc	2.28
		TTA	0.20	±	0.00	cde	0.30	±	0.00		1.48	±	0.25	abc	−1.28
		log CFU/g	7.58	±	0.54	bc	nd				9.35	±	0.01	ab	1.77
		*Growth log CFU/g*									*1.77*				
*Lp. plantarum*	**C38**														
		pH	6.75	±	0.01	abc	5.93	±	0.04	ab	4.41	±	0.04	abc	2.34
		TTA	0.25	±	0.00	abcde	0.40	±	0.00		1.18	±	0.04	abc	−0.93
		log CFU/g	7.70	±	0.33	abc	nd				9.58	±	0.02	ab	1.88
		*Average pH decrease*													2.25
		*Average TTA increase*													−1.22
		*Average growth log CFU/g*													1.47

Values are expressed as the mean ± SD from triplicate determinations. Abbreviations: *nd* Not determined. Different letters indicate statistical difference related to the different characteristics of LAB isolates (viable cell-counts, pH and total titratable acidity) at specific time (0 h, 4 h, or 7 h) using least significant difference test (*P* < 0.05). *P*-values were adjusted using Bonferroni’s method

### Effect of technological, functional and safety properties of LAB isolates

#### LAB isolates showed different abilities to biosynthesize EPS

Overall, most of the LAB strains were able to grow on maltose, glucose, sucrose, and lactose (11, 11, 10 and 9 LAB isolates, respectively, Table 3). *Lp. plantarum* A16 and B11 produced EPS using different carbon sources as shown in Table 3. *Lp. plantarum* A16, B11, B15, B27, B32, and C28 strains were only able to produce EPS while growing on starch (Table 3). Interestingly, 2 out of the 20 LAB strains did not produce EPS (*Lp. plantarum* B19 and B28, Table 3).

**Table 3:** Technological and functional properties of LAB strains isolated from soft wheat fermentations and selected for their antifungal activity

Lactic acid bacteria		Exopolysaccharides production						Xylanase activity (mm)							Amylolytic activity (mm)				Phytase activity (mm)	Proteolytic activity	
		Starch	Glucose	Lactose	Maltose	Sucrose	Frequency	Phosphate			Acetate									Casein agar	Gelatin agar
A16	*Lp. plantarum*	+	+	+	+	+	5	1.00	±	1.73	6.33	±	0.58	c	1.66	±	0.58	abc	2.0–2.5	++	
A23	*Lc. casei*	−	+	++	+	−	3	0.00	±	0.00	7.00	±	0.00	ab	1.66	±	0.58	abc	2.0–2.5	+	
A25	*Lp. plantarum*	−	+	+	+	+	4	0.00	±	0.00	7.00	±	0.00	ab	3.00	±	0.00	abc	2.0–2.5	++	
A310	*Lp. plantarum*	−	+	+	+	+	4	0.00	±	0.00	7.00	±	0.00	ab	3.33	±	0.58	ab	2.0–2.5		
B11	*Lp. plantarum*	+	+	+	+	+	5	1.33	±	2.31	7.66	±	0.58	a	4.00	±	0.00	a	2.0–2.5		
B15	*Lp. plantarum*	+	−	+	+	−	3	2.66	±	2.31	7.33	±	0.58	ab	4.00	±	0.00	a	1.5–1.9		++
B18	*Lp. plantarum*	−	+	+	−	+	3	3.33	±	0.58	7.33	±	0.58	ab	3.00	±	1.00	abc	2.0–2.5	+	
B19	*Lp. plantarum*	−	−	−	−	−	0	0.00	±	0.00	7.00	±	0.00	ab	2.66	±	0.58	abc	2.0–2.5		
B21	*Lp. plantarum*	−	+	−	−	−	1	3.66	±	0.58	6.66	±	0.58	abc	1.33	±	0.58	bc	2.0–2.5		
B23	*Lp. plantarum*	−	++	+	++	+	4	5.33	±	0.58	7.66	±	0.58	a	3.00	±	0.00	abc	2.0–2.5	+	
B27	*Lp. plantarum*	+	−	−	+	−	2	0.00	±	0.00	6.33	±	0.58	c	2.33	±	0.58	abc	2.0–2.5		
B28	*Lc. casei*	−	−	−	−	−	0	1.00	±	1.73	6.66	±	0.58	abc	2.66	±	0.58	abc	1.5–1.9		
B29	*Lp. plantarum*	−	+	−	+	+	3	1.00	±	1.73	6.66	±	0.58	abc	3.33	±	1.15	ab	2.0–2.5		
B32	*Lp. plantarum*	+	−	−	−	+	2	0.00	±	0.00	6.33	±	0.58	c	2.33	±	1.53	abc	2.0–2.5		
B33	*Lc. casei*	−	+	−	+	+	2	3.33	±	0.58	6.33	±	0.58	c	0.00	±	0.00	abc	2.0–2.5		
C11	*Lp. plantarum*	−	+	+	+	+	4	3.33	±	0.58	6.33	±	0.58	c	2.66	±	0.58	abc	2.0–2.5	++	
C12	*Lp. plantarum*	−	+	+	++	+	4	3.33	±	0.58	7.00	±	0.00	ab	2.66	±	1.53	abc	1.5–1.9		
C17	*Lp. plantarum*	−	++	−	+	−	2	3.66	±	0.58	6.66	±	0.58	abc	1.66	±	1.15	abc	2.0–2.5		
C28	*Lp. plantarum*	+	−	−	−	+	2	0.00	±	0.00	7.00	±	0.00	ab	1.00	±	0.00	c	2.0–2.5		++
C38	*Lp. plantarum*	−	−	+	+	−	2	3.66	±	0.58	7.33	±	0.58	ab	3.00	±	0.00	abc	1.5–1.9	+	
Control strain	*Amyb. amylophilus* 20,533														1.66	±	0.58	abc			
	**Frequency**	6	13	11	14	12															

Values are expressed as the mean ± SD from triplicate determinations. Different letters indicate statistical difference related to the different technological and functional properties of LAB isolates at specific time (0 h, 4 h, or 7 h) using least significant difference test (*P* < 0.05). *P*-values were adjusted using Bonferroni’s method

#### Significant differences in the xylanase activity of LAB isolates

Thirteen out of 20 LAB isolates showed an activation zone diameter ranging between 1.00 and 5.33 mm in plate assay with sodium phosphate buffer (Table 3). Interestingly, when the plates contained sodium acetate buffer, a significant difference between the xylanase activity of LAB isolates was observed (Table 3, *P* < 0.05). In detail, significantly higher xylanase activity was detected in *Lp. plantarum* B11 and B23, reaching an activity zone diameter of 7.66 mm, while the lowest activity was observed in *Lp. plantarum* A16, B27, B32, B33, C11, in which the activity zone diameter reached 6.33 mm (*P* < 0.05, Table 3).

#### LAB isolates exhibited a significant different amylolytic activity

A significantly higher amylolytic activity was detected in *Lp. plantarum* B11 and B15, reaching an activity zone diameter of 4 mm, while the lowest activity was observed in *Lp. plantarum* C28, in which the activity zone diameter reached 1 mm (*P* < 0.05, Table 3). Overall, the amylolytic activity was displayed by all LAB isolates, except for *Lc. casei* B33 (Table 3).

#### LAB phytases are apparently not affected by LAB diversity

The ability to hydrolase hexacalcium phytate was present among all the LAB isolates tested. Overall, sixteen out of 20 LAB isolates exhibited a high inhibiting zone diameter, reaching 2.0–2.5 mm (Table 3).

#### Casein agar plates induced a higher number of proteolytic reactions from LAB isolates

Only two LAB isolates were able to degrade gelatin in plate assays as observed by the formation of a clear halo after incubation on a medium containing gelatin (*Lp. plantarum* B15 and C28, Table 3). In contrast, when using casein agar, seven LAB isolates were able to degrade casein in plate assay (*Lp. plantarum* A16, A25, B18, B23, C11, C38 and *Lc. casei* A23, Table 3).

#### Selected LAB isolates displayed single, double and multiple antibiotic resistance

Ampicillin resistance was identified for all LAB isolates regardless of the time of incubation, except for one strain (*Lc. casei* A23, Table 4). Interestingly, five LAB isolates displayed a double antibiotic susceptibility after 24 h (Table 4) while, after 48 h, all LAB isolates were resistant to Amp and Cli. Multiple antibiotic resistance was detected in 3 LAB isolates after 48 h (Table 4).

**Table 4:** Antibiotic resistance of LAB strains selected for their antifungal, technological and functional activities

Lactic acid bacteria		Antibiotic resistance	
		24 h	48 h
A16	*Lp. plantarum*	Amp/Cli	Amp/Cli
A23	*Lc. casei*	Ery/Tet	Ery/Cli/Tet
A25	*Lp. plantarum*	Amp	Amp/Cli
A310	*Lp. plantarum*	Amp/Cli	Amp/Gen/Cli
B11	*Lp. plantarum*	Amp/Cli	Amp/Cli
B15	*Lp. plantarum*	Amp	Amp/Cli
B18	*Lp. plantarum*	Amp/Cli	Amp/Cli/Chl

LAB with MIC higher than EFSA breakpoints are considered as resistant strains

## Discussions

*A. niger* is a spoilage fungus known to grow rapidly on the surface of bakery products (Smith, Daifas, El-Khoury, Koukoutsis, & El-Khoury, 2004). Previous studies have shown that different LAB strains (*Lp. plantarum* CE42, CE60, CE84, *Ll. curvatus* CE83 and *Pediococcus pentosaceus* CE65 and CE23), isolated from wheat bran sourdough, have a strong antifungal activity towards *A. niger* and *Aspergillus oryzae* (Manini et al. 2016). In this regard, our study showed the ability of *Lp. plantarum* isolates to inhibit *in vitro* the growth not only of *Aspergillus* species but also of a broad range of filamentous fungi (16 molds tested) which were chosen for their spoilage potential in bread and bakery goods, and/or for their potential to produce mycotoxins.

The antifungal potential differed between LAB isolates, and our results showed that the inhibition of different fungal species was strain dependent. Similar inhibition activity was observed elsewhere in *Ll. brevis* but not in *Lp. plantarum* (Manini et al. 2016; Ruiz Rodríguez et al. 2016). However, the antifungal effect strain dependency of *Lp. plantarum* has been observed from other authors in isolates from different food matrices (Russo et al. 2017). In terms of biocontrol potential, the application of different LAB strains in food processing could expand horizons for preventing food spoilage. The capability of *Lp. plantarum* to prevent spoilage has been linked to the direct competition for growth substrates between this bacteria and spoilage fungi, and to the production of compounds such as lactic acid, acetic acid, hydrogen peroxide, phenyllactic acid and bacteriocins (Cortés-Zavaleta et al. 2014; Gupta and Srivastava 2014). In the present research, the nature of LAB antifungal strenght (organic acid and/or proteinaceous metabolites) was not investigated. Some authors highlighted the major contribute of organic acids for antifungal activity in LAB strains and identified phenyllactic acid as one of the most effective antifungal compounds in *Lp. plantarum*, showing that its production is strain-dependent (Dal Bello et al. 2007; Schnurer and Magnussoon 2007). The disadvantages of studying antifungal activity based on colony growth in synthetic selective media are well known and include that the concentration of glucose seems to affect antifungal activity and MRS medium could stimulate the production of antifungal compounds (Fraberger et al. 2020). Further research is needed to assess the impact of using LAB strains as a starter cultures in sourdough as antifungal controlling agents.

The ability of LAB to decrease the pH during sourdough fermentation is an important technological feature of this bacterial group. Recently, differences in the growth and acidification rate between different LAB strains have been reported (Manini et al. 2016; Ruiz Rodríguez et al. 2016). In this study, we also observed a significant difference in LAB growth and acidification rate between isolates, further confirming previous study observation. Noteworthy, LAB isolates showed lower population growth (final LAB growth range between 8.30 and 9.89 log CFU/g range) compared with previous researches (11 log CFU/g) (Manini et al., 2016). The capability of LAB isolates to reduce the pH to about 4, after 7 or 8 h, is an important characteristic to obtain bread and bakery products with better rheological and flavor characteristics. In our study, the highest pH values were showed particularly by *Lc. casei* strains, as observed by other authors (Paucean et al. 2013).

Concerning the technological properties, such as EPS and enzymatic activities, of the LAB isolates. EPS such as glucans and/or fructans play an important role in the texture, taste perception and stability of fermented food (Tieking and Gänzle 2005; Galle and Arendt 2014). Previous studies suggested that at least one or more EPS producing bacterial strain is found in sourdough microbiota (Tieking, et al. 2003; Manini et al. 2016). According to Manini et al. (2016), one of the major EPS producers was *Lp. plantarum*. The differences in EPS production found in our study between LAB isolates indicates that EPS production is largely strain-dependent. On the other hand, xylanase and phytase are used to increase dough viscosity, bread volume, shelf life and improve mineral bioavailability in bakery products (Poutanen et al. 2009). The ability of all LAB isolates tested in our study to degrade hexacalcium phytate is consistent with previous studies (De Angelis et al. 2003; Manini et al. 2016).

The degradation of peptides and proteins by LAB contributes to flavor, antimicrobial activity, and structure of different foods. In our study, the proteolysis evaluation resulted in scarce protein degradation, in agreement with previous findings. Only a LAB strain belonging to *Lactococcus lactis* species and isolated from quinoa sourdough fermentation showed strong proteinase activity (Ruiz Rodríguez et al. 2016). Furthermore, our results indicate different proteolytic capacities between LAB isolates, in accordance elsewhere (Alfonzo et al. 2013). Differences in the ability to utilize gelatin or casein may be related to the differences in the substrate specificities of the enzyme produced. A comparative genome analysis of several *Lp. plantarum* strains demonstrated the versatility to acquire and retained functional capacities (Siezen et al. 2010) but lack of environmental adaptation (Martino et al. 2016). Recently, the capacity of *Lp. plantarum* isolated from cassava to synthesize EPS, metabolize carbohydrate (starch), polyphenols and vitamin B has been demonstrated through genomics and transcriptomics (Turpin et al. 2018). Further research is needed to find the link between gene content and functional properties of different LAB strains in food and their interactions.

The microbial resistance to antibiotics represents a concern for public health. *Lp. plantarum* and *Lc. casei* here tested are included in the Qualified Presumption of Safety (QPS) list of the European Food Safety Authority (EFSA 2007). However, all *Lp. plantarum* isolates assayed for antibiotics sensitivity, showing susceptibility to ampicillin and clindamycin after 48 h, contrarily to what described for *Lp. plantarum* strains isolated from quinoa sourdoughs and wheat bran elsewhere (Manini et al. 2016; Ruiz Rodríguez et al. 2016). Noteworthy, among *Lp. plantarum* we observed that two isolates showed an antibiotic multi-resistance. Differences in the antibiotic resistance between LAB isolates may be attributed to the non-specific mechanism of multidrug transporters, general stress-induced response, a mutation on penicillin-binding proteins (Delgado et al. 2005), or defective autolytic enzyme activity (Kim et al. 1982). In our study, two LAB isolates were susceptible to chloramphenicol, erythromycin, and tetracycline, confirming that lactobacilli are usually resistant to antibiotics that inhibit the synthesis of proteins (Danielsen and Wind 2003; Shao et al. 2015). Further investigation is needed to explore if this antibiotic resistance is non-transmissible.

## Conclusions

In conclusion, the antifungal activity of the different LAB strains isolated from type “0” soft wheat flour sourdough inhibited the growth of several fungal species *in vitro*. The LAB strains used in this study demonstrated a different antifungal potential, EPS production, and enzymatic activity. *Lp. plantarum* A16, A25, B11, and B15 reduced the growth of several fungal species and showed the greatest capabilities to growth in different carbon sources. These results demonstrated that type “0” soft wheat flour sourdough is a good source to isolate LAB strains with potential for future food applications, as natural bio-control agents to inhibit fungal growth and most probably to extend the shelf life of bakery products. Care should be taken with the selection of LAB strains resistant to ampicillin and clindamycin. Further study is recommended to optimize the conditions to increase the antifungal activity for food application in greening the food processing industry.

## Data Availability

The authors declare data transparency.
